# Malignant peripheral nerve sheath tumor of the nasal cavity: a rare diagnostic and therapeutic challenge

**DOI:** 10.1093/jscr/rjaf349

**Published:** 2025-05-30

**Authors:** Amine Oussalem, Anas Azgaoui, Bouchra Dani, Malik Boulaadas

**Affiliations:** Department of Maxillo-Facial Surgery, Avicenne Hospital, Area Lamfadel Cherkaoui Rabat – Institut, Rabat BP 6527, Morocco; Department of Maxillo-Facial Surgery, Avicenne Hospital, Area Lamfadel Cherkaoui Rabat – Institut, Rabat BP 6527, Morocco; Department of Maxillo-Facial Surgery, Avicenne Hospital, Area Lamfadel Cherkaoui Rabat – Institut, Rabat BP 6527, Morocco; Department of Maxillo-Facial Surgery, Avicenne Hospital, Area Lamfadel Cherkaoui Rabat – Institut, Rabat BP 6527, Morocco

**Keywords:** malignant peripheral nerve sheath tumor, nasal cavity, surgical resection, chemotherapy, rare sarcoma

## Abstract

Malignant peripheral nerve sheath tumors (MPNSTs) are rare, aggressive sarcomas, often affecting the head and neck, with higher incidence in neurofibromatosis type 1. Early diagnosis is crucial to prevent recurrence and metastasis. We report a 78-year-old male with a rapidly enlarging right nasal cavity mass over 4 months, associated with recurrent epistaxis. His medical history included diabetes, hypertension, and tobacco use. Examination revealed a firm, non-adherent mass obstructing the nasal cavity. Computed tomography (CT) imaging localized it to the right inferior turbinate. Due to size and location, biopsy was not performed. Surgical resection confirmed a high-grade MPNST (FNCLCC grade 3). Chemotherapy was initiated postoperatively, but the patient passed away soon after, highlighting the tumor’s aggressiveness. MPNSTs should be considered in nasal mass differentials, especially in elderly smokers. Early surgery and chemotherapy may improve outcomes, emphasizing the need for a multidisciplinary approach.

## Introduction

Malignant peripheral nerve sheath tumors (MPNSTs) are rare and aggressive sarcomas that arise from peripheral nerves or surrounding structures [1]. Their nonspecific symptoms often make them difficult to diagnose, particularly in the head and neck, where they account for a small portion of soft tissue cancers [2]. Although these tumors are commonly associated with neurofibromatosis type 1 (NF1), they can also occur sporadically in individuals without any genetic predisposition [3]. MPNSTs are highly prone to recurrence and metastasis, typically spreading to the lungs and bones [4]. However, their subtle clinical signs often lead to delayed diagnosis [5]. Imaging techniques such as computed tomography (CT) and magnetic resonance imaging (MRI) play a critical role in preoperative assessment, but histopathological examination remains the gold standard for diagnosis [6]. Surgical resection is the main treatment approach for MPNSTs, though the role of chemotherapy and radiotherapy is still debated and should be tailored to each case based on tumor grade and location [7]. This case report focuses on a rare MPNST in the nasal cavity, emphasizing the importance of early diagnosis, timely treatment, and a multidisciplinary approach to improve patient outcomes [8].

## Case report

A 78-year-old male was referred to the Oral and Maxillofacial Surgery Department at Ibn Sina Hospital, Rabat, due to a rapidly growing mass in the right nasal cavity, accompanied by recurrent epistaxis. His medical history included well-controlled diabetes, hypertension, and active tobacco use.

**Figure 1:**
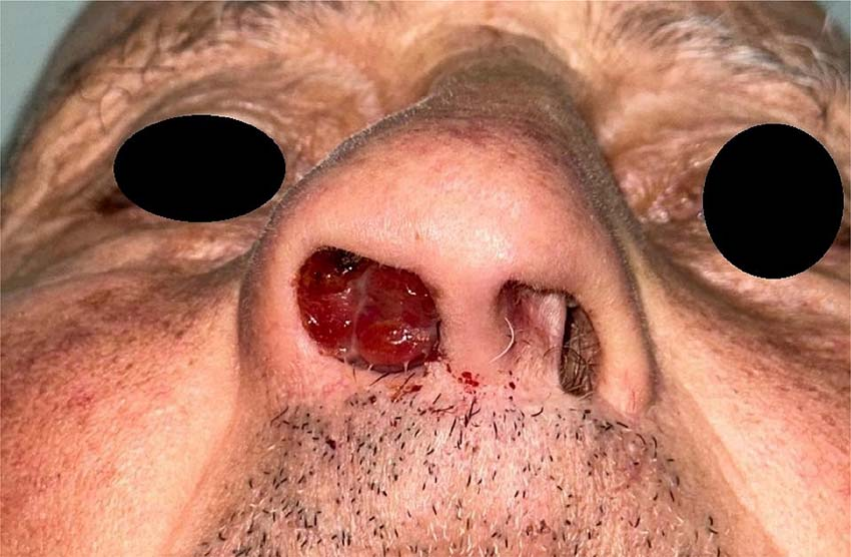
Image showing the macroscopic aspect of the tumor.

On examination, a firm, non-adherent, painless mass was found obstructing the right nasal cavity. Measuring approximately 3 cm, it caused significant airflow reduction and bled upon contact (Fig. 1).

**Figure 2:**
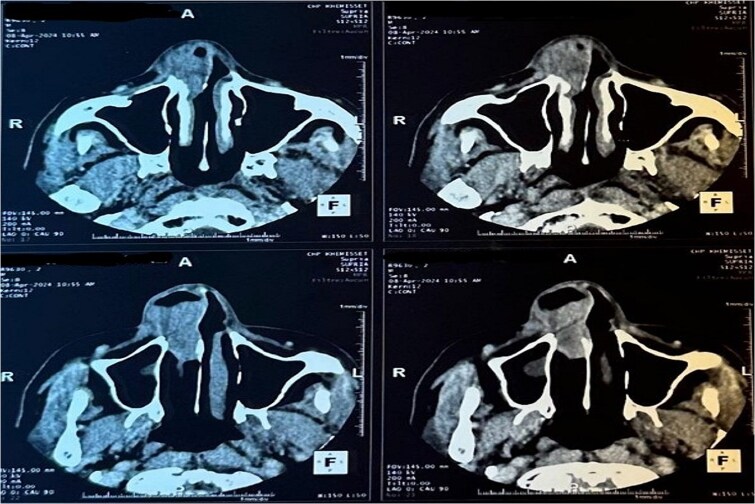
Tomography image of the paranasal sinuses in axial slice with parenchymal window revealing a tumoral-like mass in the right nasal cavity, centered on the right inferior turbinate.

**Figure 3:**
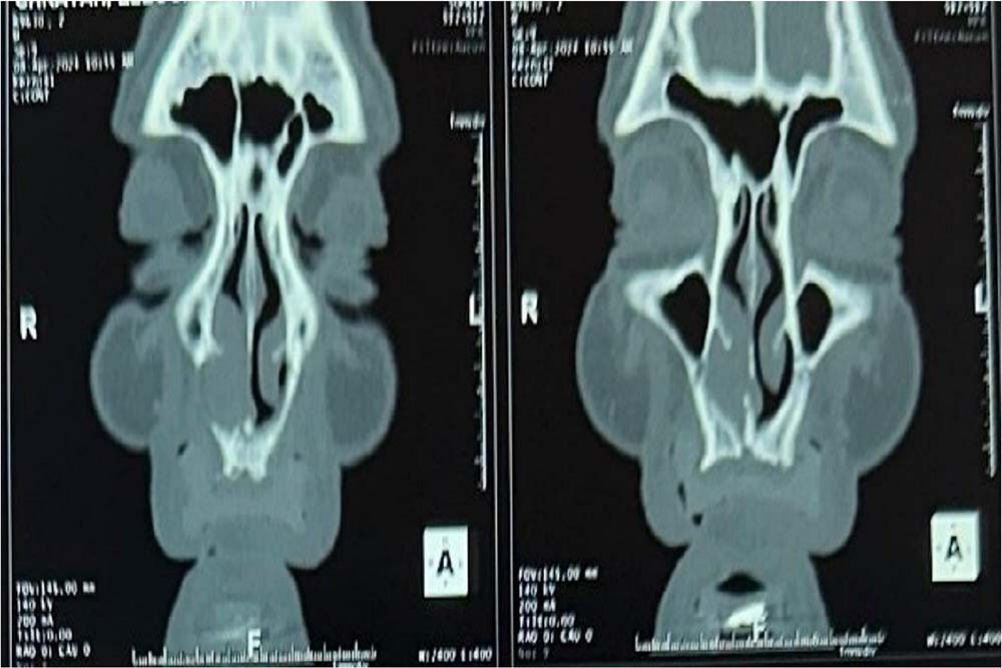
Tomography image of the paranasal sinuses in coronal slice with parenchymal window revealing a tumoral-like mass in the right nasal cavity, centered on the right inferior turbinate.

A CT scan revealed a mass centered on the right inferior turbinate, with no bony involvement but a polypoid filling of the right maxillary sinus, raising concern for malignancy (Figs 2 and 3).

Due to the tumor’s size and suspicion of malignancy, a biopsy was not performed, and surgical resection was planned. The procedure, done via a paralatéronasale approach, confirmed a high-grade, poorly differentiated spindle cell sarcoma, which was classified as a grade 3 MPNST upon histopathological analysis.

**Figure 4:**
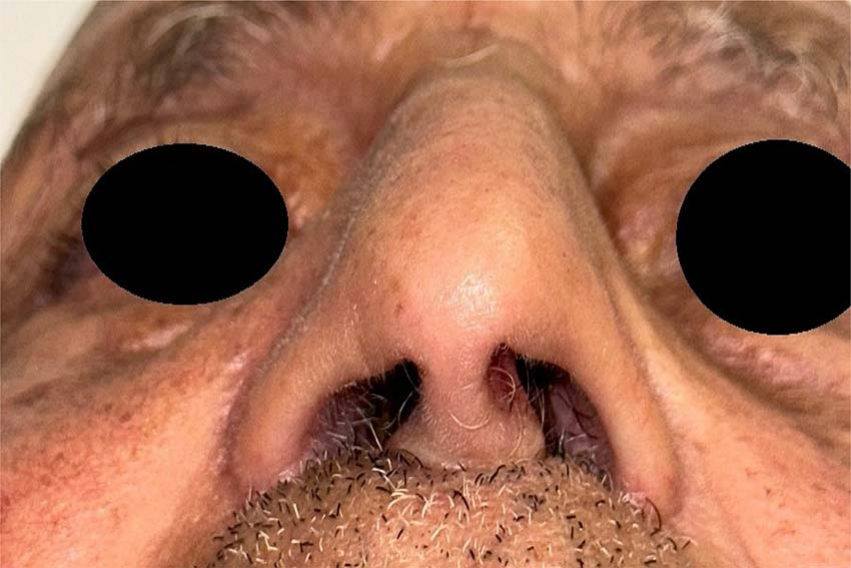
Postoperative image of the patient 1 month after surgery.

One month after surgery, chemotherapy was initiated (Fig. 4).

Unfortunately, the patient passed away shortly after the first sessions, reflecting the aggressive nature of the sarcoma.

## Discussion

Diagnosing MPNSTs is particularly challenging due to their rarity, aggressive behavior, and nonspecific clinical presentation. MPNSTs are most commonly linked with NF1, but sporadic cases, like the one presented here, also occur [1, 2]. These tumors arise primarily from Schwann cells, though some can originate from perineural fibroblasts [3]. MPNSTs in the head and neck are exceptionally rare and often present with symptoms that resemble benign conditions or other types of tumors. Clinical features such as epistaxis, unilateral nasal obstruction, and facial swelling can be mistaken for less serious issues [4].

Imaging, particularly MRI, plays a key role in evaluating soft tissue tumors. However, CT scans, as used in this case, are also valuable for determining tumor size, location, and the involvement of adjacent structures. While MRI would have provided better soft tissue differentiation and clearer views of the tumor’s relationship to surrounding nerves, both CT and MRI are indispensable for guiding surgical planning [5, 6].

Histopathological examination typically reveals spindle-shaped cells with high mitotic activity arranged in fascicles, but the findings can be nonspecific. Immunohistochemistry (IHC), particularly for S-100 protein, is essential for confirming the diagnosis, as it helps distinguish MPNSTs from other spindle cell tumors, such as fibrosarcomas and fibrous histiocytomas [7]. In large or high-risk tumors, direct surgical resection is sometimes preferred over biopsy to reduce the risk of tumor seeding.

Surgical excision remains the mainstay of treatment for MPNSTs, with the extent of resection being a critical factor for prognosis. In this case, resection was performed using a paralatéronasale approach to ensure clear margins and minimize the risk of recurrence. Adjuvant chemotherapy, which is typically recommended for high-grade tumors, remains a subject of debate, particularly for head and neck MPNSTs [8]. Chemotherapy, often involving agents like doxorubicin and ifosfamide, is commonly used for high-grade or metastatic tumors [6]. Unfortunately, chemotherapy did not prevent recurrence in this patient, and he passed away shortly after beginning treatment.

The high recurrence rates of MPNSTs, even with clear margins, underscore the importance of a multidisciplinary approach to management. This case highlights the need to consider MPNSTs in the differential diagnosis of nasal masses, particularly in elderly patients with risk factors such as tobacco use. A combined approach of early diagnosis surgical resection and chemotherapy, guided by a multidisciplinary team, offers the best chance for favorable outcomes [9, 10].

## Conclusion

MPNSTs present a significant diagnostic challenge due to their often subtle clinical presentation and the variety of associated symptoms. This case of an MPNST located in the nasal cavity highlights the importance of thorough preoperative evaluation, including imaging and histopathological criteria, to establish an accurate diagnosis before proceeding with treatment. Surgery remains the cornerstone of management, but the role of adjuvant therapies, such as chemotherapy, warrants careful consideration based on tumor aggressiveness and location. After 6 months of follow-up, the absence of recurrence in our case suggests that a multimodal approach, combining surgical resection and chemotherapy, can yield favorable outcomes even in complex clinical scenarios. Management of MPNSTs requires a multidisciplinary approach and rigorous follow-up to ensure the best possible results and minimize the risk of recurrence or metastasis.
